# Normative data for middle-aged Brazilians in the Mattis Dementia Rating Scale

**DOI:** 10.1590/1980-57642020dn14-040004

**Published:** 2020-12

**Authors:** Guilherme Almeida Carvalho, Paulo Caramelli

**Affiliations:** 1 Rede Sarah de Hospitais de Reabilitação – Belo Horizonte, MG, Brazil.; 2 Programa de Pós-Graduação em Ciências Aplicadas à Saúde do Adulto, Faculdade de Medicina, Universidade Federal de Minas Gerais – Belo Horizonte, MG, Brazil.; 3 Grupo de Pesquisa em Neurologia Cognitiva e do Comportamento, Departamento de Clínica Médica, Faculdade de Medicina, Universidade Federal de Minas Gerais – Belo Horizonte, MG, Brazil.

**Keywords:** mental status and dementia tests, education, Brazil, reference standards, middle aged, testes de estado mental e demência, educação, Brasil, padrões de referência, pessoas de meia-idade

## Abstract

Despite the advances in the diagnosis of dementia, neuropsychological assessment remains an important tool. The Mattis Dementia Rating Scale (DRS) was designed to evaluate people with suspected dementia and allows for the analysis of different cognitive domains. Considering the numerous cases of early-onset dementia, specific reference standards aimed at the middle-aged population are necessary.

**Objective:**

To provide normative data for the middle-aged Brazilian population in DRS and to investigate the influence of education level, age, sex, and intelligence quotient (IQ) on the results.

**Methods:**

Overall, 120 healthcare professionals and caregivers from a hospital, who were healthy, aged between 45 and 64 years, and had at least four years of formal education, were included in the study. They were equally divided into six groups. In each age group (45–54 and 55–64 years), there were three educational levels: 4–7, 8–11, and 12 or more (12+) years of formal education. The results are presented as mean values, standard deviations, and percentiles. Comparisons between groups were carried out for age, sex, and education level. Age, years of formal education, and IQ were also analyzed as continuous variables by Spearman's correlation.

**Results:**

Concerning education level, the comparison between groups showed differences in the results for the total scale and subscales, except for the Construction subscale. No differences were found for age and sex. Correlations observed for years of formal education and IQ were similar. No correlation was found for age.

**Conclusions:**

The present study contributes to the evaluation of dementia concerning people younger than 65 years of age and reinforces the importance of education in the interpretation of the scores.

## RESUMO.

Apesar dos avanços no diagnóstico de demência, a avaliação neuropsicológica continua sendo uma importante ferramenta. A Escala de Avaliação de Demência de Mattis (*Mattis Dementia Rating Scale* — DRS) foi projetada para avaliar pessoas com suspeita de demência e permite a análise de diferentes domínios cognitivos. Dados os numerosos casos de demência com início precoce, são necessárias normas específicas para a população de meia-idade.

**Objetivo:**

Fornecer dados normativos para a população brasileira de meia-idade na escala Mattis e investigar a influência da escolaridade, idade, sexo e quociente de inteligência (QI) nos resultados.

**Métodos:**

120 funcionários e cuidadores saudáveis de um hospital, com idade entre 45 e 64 anos e com pelo menos quatro anos de escolaridade foram incluídos no estudo, os quais foram divididos igualmente em seis grupos. Havia três níveis educacionais para cada faixa etária (45–54 e 55–64 anos): 4 a 7 anos de estudo (4–7), 8 a 11 (8–11) e 12 anos ou mais (12+). Os resultados são apresentados como valores médios, desvios padrão e percentis. Foram realizadas comparações entre os grupos de acordo com idade, sexo e escolaridade. Idade, anos de estudo e QI foram também analisados como variáveis contínuas através da correlação de Spearman.

**Resultados:**

Para a educação, a comparação entre os grupos mostrou diferenças nos resultados da escala total e subescalas, exceto na subescala Construção. Não foram identificadas diferenças para idade e sexo. As correlações observadas para anos de estudo e QI foram semelhantes. Nenhuma correlação foi encontrada para idade.

**Conclusão:**

O presente estudo contribui para a avaliação da demência em indivíduos com menos de 65 anos e reforça a importância de considerar o nível educacional na interpretação dos escores.

## INTRODUCTION

Despite the many advances in the diagnosis of dementia, neuropsychological assessment remains an essential tool in this process.^1^ Some of the most popular neuropsychological test batteries used to detect dementia, such as the Mini-Mental State Examination (MMSE) and the Mini-Cog, are brief.^2^ They are useful for dementia screening, but have limitations in the adequate characterization of neuropsychological profiles and dementia staging.

Conversely, a comprehensive neuropsychological assessment requires many sessions, and some tasks can be difficult for moderate or severe stages of cognitive impairment. The Mattis Dementia Rating Scale (DRS) is a neuropsychological test battery designed for people with known or suspected dementia.^3^ The tasks are suitable for people in different dementia stages, allowing for the analysis of specific cognitive domains and the follow-up of cognitive functioning over time. The evaluation of patients with dementia carried out with DRS usually lasts 30 to 45 minutes.

The total scale is divided into five subscales: Attention (ATT), Initiation/Perseveration (I/P), Construction (CONST), Conceptualization (CONCEPT), and Memory (MEM). This division enables the identification of different cognitive profiles in distinct forms of dementia, thus contributing to a differential diagnosis. When different dementing disorders were compared, studies showed comparable total scores, but differences on subscales. Patients with Alzheimer's disease (AD) score worse on the MEM subscale than patients with other disorders such as frontotemporal dementia,^4^ vascular dementia,^5^ Huntington's disease, or progressive supranuclear palsy.^6^ On the other hand, patients with AD had better performance on I/P.

In Brazil, Porto et al.^7^ ^,^ ^8^ and Foss et al.^9^ ^,^ ^10^ conducted studies to adapt the scale and define standards for the Brazilian population. These studies confirm international findings regarding the influence of age and education on the results. Strauss et al. recommend that normative data be classified according to age and education level.^3^

Most cases of dementia begin after the age of 65.^11^ However, some cases present an early onset, indicating that normative data for this population are necessary. In a population-based study aiming to investigate the prevalence of dementia in a small Brazilian city, César found a prevalence of 5.3% among individuals aged 60 to 64 years, which is five times higher than in other regions worldwide. The author stated that this finding is probably related to low educational levels.^12^

In the normative study conducted by Foss et al. on 502 Brazilians, there were fewer participants under the age of 60. Thus, the authors recommended additional studies with more participants in the age group of 50–60 years.^10^

The present study aimed to provide normative data for individuals aged 45 to 64 years and to investigate the influence of education level, age, sex, and intelligence quotient (IQ) on results of the DRS.

## METHODS

A more detailed description of the methods can be found on a previous study.^13^

### Participants

The sample consisted of 120 healthcare professionals and caregivers from a hospital, who were cognitively-healthy, aged between 45 and 64 years, had at least four years of formal education, and were randomly selected. They were equally divided into six groups according to age and education level. In each age group (45–54 and 55–64 years), there were three education levels: 4–7, 8–11, and 12 or more (12+) years of formal education.

Individuals with neurological and psychiatric disorders whose symptoms could cause cognitive impairment at the time of the tests were excluded as well as subjects with hearing or visual impairment. Moreover, individuals who reported the use of psychoactive drugs within three weeks prior to the administration of the tests were excluded. Subjects who reported alcohol dependence or who were using illicit drugs were also excluded.

### Setting

The study was approved by the Ethics Committees of Hospital Sarah and Universidade Federal de Minas Gerais. The first author, who has considerable experience with neuropsychological examination, collected data in an appropriate room at Hospital Sarah in Belo Horizonte (state of Minas Gerais), Brazil, between May 2015 and October 2016.

### Instruments

The MMSE was used as a study entry criterion. Individuals below the established education-adjusted cutoff points were excluded (24 for 4–7 years of formal education, and 26 for 8 or more years).^14^ ^,^ ^15^ Modules A, J, and O of the Mini-International Neuropsychiatric Interview (MINI) were also performed to investigate frequent psychiatric disorders.^16^ ^,^ ^17^ Individuals who met the criteria for major depressive disorder, generalized anxiety disorder, or alcohol dependence, according to MINI, were excluded from the study. Participants also completed the two-subtest form of the Wechsler Abbreviated Scale of Intelligence (WASI) to determine their IQ.^18^

DRS has a total score of 144, which is divided among the five subscales, namely: 37 points related to the ATT subscale; 37, to I/P; six, to CONST; 39, to CONCEPT; and 25, to MEM.^7^ Table 1 shows a brief description of the subtests in the subscales.^3^ ^,^ ^7^

**Table 1:** Brief description of the Mattis Dementia Rating Scale subtests

Subscale	Subtests	Maximum points
Attention	Digit span	37
	Commands	
	Imitation	
	Letter A counting (two tasks)	
	Word list reading	
	Visual matching	
Initiation/ Perseveration	Verbal fluency tests (supermarket products and body parts)	37
	Sound repetition	
	Alternating movements	
	Graphomotor design	
Construction	Copying designs	6
Conceptualization	Similarities	39
	Inductive reasoning	
	Differences	
	Similarities – Multiple choice	
Memory	Orientation	25
	Verbal recall – reading and sentence generated by the examinee	
	Verbal recognition (word list reading from the Attention subscale)	
	Visual recognition (visual matching from the Attention subscale)	
Total score		144

Sources: Scoring Booklet* and Strauss et al.^3^

* The scoring booklet of the Brazilian adaptation was kindly provided by the Department of Neurology - Hospital das Clínicas da Faculdade de Medicina da Universidade de São Paulo.^7^

### Procedures

In the first session, after signing the informed consent form, the participants attended an interview and completed the MMSE and the MINI. In a second session, the application of the WASI and DRS was carried out.

In the interview, age, years of formal education, history of possible neurological and psychiatric illnesses, health conditions, medications in use, and possible history of consumption of alcoholic beverages and drug use were verified.

### Statistical analysis

The Shapiro-Wilk test was used to verify the hypothesis of normal distribution. Nonparametric tests were the most appropriate for the analyses.

The Kruskal-Wallis test (K-W) was chosen to compare the groups according to education level, and the Mann-Whitney (M-W) test was used for*post hoc* analyses with a Bonferroni correction for p-values. The Mann-Whitney test was used in paired comparisons to analyze sex and age. Age, years of formal education, and IQ were also analyzed as continuous variables with Spearman's correlation (r_s_). The classification suggested by Siqueira and Tibúrcio was followed to interpret the correlations: 0–0.4, weak; 0.4–0.7, moderate; and 0.7–1.0, strong.^19^

All analyses were performed using the*Statistical Package for the Social Sciences* (SPSS) software, version 20.0. The considered level of significance was p<0.05. In cases in which the Bonferroni correction was performed, p<0.017 was considered significant.

## RESULTS

Overall, 153 individuals were invited to participate in the study. There were 13 nonrespondents and 18 exclusions: 12 due to the use of medications with potential negative cognitive effects; one due to neurological disease; one for being outside the age group; and four for failing to meet the diagnostic criteria of the MINI interview. Of the 122 participants included, two withdrew during the second stage, in which the WASI and DRS were applied.

Table 2 shows the sociodemographic characteristics of the participants. Results of DRS are presented as mean values, standard deviations, and percentiles (Table 3). Considering the non-normal frequency distribution, the use of percentiles (P) was recommended.

**Table 2:** Sociodemographic characteristics of the participants

Groups (years of formal education/age)	Age (years)^a^	Years of formal education^1^	Sex^b^	IQ^a^	n
4–7/45–64	49.9±3.1	5.2±1.1	10 (50%)	81.4±14.2	20 (16.7%)
4–7/55–64	59.3±2.9	5.0±1.4	13 (65%)	77.2±9.5	20 (16.7%)
8–11/45–64	49.7±2.5	10.7±0.8	11 (55%)	96.5±11.2	20 (16.7%)
8–11/55–64	57.9±1.8	10.1±1.3	15 (75%)	91.9±16.5	20 (16.7%)
≥12/45-64	48.8±2.8	15.6±2.4	15 (75%)	110.9±11.1	20 (16.7%)
≥12/55-64	58.5±2.3	16.9±2.7	15 (75%)	111.3±10.5	20 (16.7%)

Source: Prepared by the authors.

a Mean values±standard deviation;

b percentage of females; IQ: intelligence quotient.

**Table 3:** Normative data in the Mattis Dementia Rating Scale

Age (years)		45 to 54			55 to 64		
Education level (years)		4–7	8–11	12+	4–7	8–11	12+
Total score							
	Mean values±SD	128.7±7.6	135.9±4.8	138.9±3.2	128.6±5.6	132.2±5.2	139.2±3.4
	P90	139	141	143	136	140	143
	P75	134	139	141	134	138	142
	Median	130	137	140	128	131	140
	P25	126	134	136	124	128	133
	P10	114	127	134	121	126	133
Attention							
	Mean values±SD	35.9±1.0	36.2±1.3	36.5±0.7	35.5±1.0	35.7±1.3	36.7±0.6
	P90	37	37	37	37	37	37
	P75	37	37	37	36	37	37
	Median	36	37	37	36	36	37
	P25	35	35	36	35	35	36
	P10	34	34	35	34	33	36
Initiation/Perseveration							
	Mean values±SD	32.8±4.1	35.9±1.8	36.7±0.7	34.5±2.1	34.7±3.1	36.2±1.6
	P90	37	37	37	37	37	37
	P75	37	37	37	36	37	37
	Median	33	37	37	35	36	37
	P25	31	36	36	33	32	36
	P10	26	32	35	31	31	33
Construction							
	Mean values±SD	5.7±1.1	6.0±0.2	6.0±0.0	5.5±1.1	6.0±0.2	6.0±0.0
	P90	6	6	6	6	6	6
	P75	6	6	6	6	6	6
	Median	6	6	6	6	6	6
	P25	6	6	6	6	6	6
	P10	3	6	6	3	6	6
Conceptualization							
	Mean values±SD	31.1 ±4.1	34.7±2.9	35.6± 2.2	29.8±5.0	32.8±4.2	36.2±2.7
	P90	37	38	38	37	38	39
	P75	34	37	37	33	37	38
	Median	31	36	36	31	34	37
	P25	28	33	34	26	29	35
	P10	26	29	31	23	27	31
Memory							
	Mean values±SD	23.3±1.4	23.2±1.5	24.2±1.1	23.4±1.3	23.1±1.5	24.3±1.0
	P90	25	25	25	25	25	25
	P75	24	24	25	24	24	25
	Median	24	24	25	23	23	25
	P25	22	23	23	23	22	24
	P10	21	22	22	21	21	22

Source: prepared by the authors.

SD: standard deviation; P: percentile; n=20 for each group.

### Education level

Analyses between groups showed significant differences for total and subscale scores (p<0.001), except for the CONST subscale (Table 4).

**Table 4:** Comparisons between groups according to education level in the Mattis Dementia Rating Scale

Education level (years)	4–7	8–11	≥12	
Results	Mean value±SD Median (Q1;Q3)	Mean value±SD Median (Q1;Q3)	Mean value±SD Median (Q1;Q3)	p–value
Total score	128.60±6.62 128.5 (125.25;133.50)	134.03±5.26 135.50 (129.00;138.75)	139.00±3.25 140.00 (137.00;141.00)	<0.001
Attention	35.68±1.00 36.00 (35.00;36.00)	35.93±1.31 36.00 (35.00;37.00)	36.55±0.64 37.00 (36.00;37.00)	<0.001
Initiation/perseveration	33.63±3.30 34.50 (31.25;36.00)	35.28±2.57 36.50 (34.00;37.00)	36.40±1.24 37.00 (36.00;37.00)	<0.001
Construction	5.58±1.06 6.00 (6.00;6.00)	5.95±0.22 6.00 (6.00;6.00)	–	–
Conceptualization	30.43±4.56 31.00 (27.00;34.00)	33.73±3.71 35.00 (31.25;37.00)	35.85±2.49 36.50 (35.00;37.75)	<0.001
Memory	23.30±1.32 24.00 (23.00;24.00)	23.15±1.46 23.00 (23.00;24.00)	24.20±1.04 25.00 (23.25;25.00)	<0.001

Source: prepared by the authors.

SD: standard deviation; n=40 for each group.

*Post hoc* analyses showed a difference in paired comparisons among the three education levels on total scores and the CONCEPT subscale. In the I/P group, differences were observed in the comparison of the 4–7 group with the 8–11 and 12+ groups. The MEM subscale evidenced differences between the 8–11 and 12+ groups as well as between the 4–7 and 12+ groups.

There were positive correlations between years of formal education and results for the total and subscale scores. Correlations were strong for the total score, moderate for the CONCEPT and I/P subscales, and weak for ATT, MEM, and CONST subscales (Table 5).

**Table 5:** Spearman's correlation (r_s_) for years of formal education, intelligence quotient, and age.

	Years of formal education	p-value	IQ	p-value	Age	p-value
Total scale	0.704	<0.001	0.812	<0.001	-0.132	=0.151
Attention	0.373	<0.001	0.492	<0.001	-0.130	=0.158
Initiation/perseveration	0.463	<0.001	0.455	<0.001	-0.044	=0.635
Construction	0.243	=0.007	0.287	=0.001	-0.067	=0.469
Conceptualization	0.604	<0.001	0.730	<0.001	-0.105	=0.254
Memory	0.316	<0.001	0.361	<0.001	-0.001	=0.988

Source: prepared by the authors.

IQ: intelligence quotient; n=120.

### Age, sex, and intelligence quotient

Age and sex did not influence the performance on DRS in paired comparisons. Similarly, no correlations were verified between DRS results and these factors.

Conversely, IQ significantly influenced performance. Positive correlations were strong for the total score and the CONCEPT subscale, whereas correlations were moderate for the ATT and I/P subscales and weak for the CONST and MEM subscales.

## DISCUSSION

The present study contributes to normative data for middle-aged Brazilians in the DRS. As discussed by Foss et al.,^10^ studies on Brazilians within this age group are necessary. As aforementioned, the results of this study confirm the influence of education level on the performance on DRS.^3^ ^,^ ^8^ ^–^ ^10^ ^,^ ^20^ ^,^ ^21^ Regarding the subtests, this influence was stronger on the CONCEPT subscale and moderate on the I/P and ATT subscales.

In the CONCEPT subscale, there are essentially verbal and nonverbal tasks demanding analogies and classification. Such skills are often developed in school, justifying greater ability for people with higher education level. The I/P subscale is mainly composed of fluency tests. The education level also contributes to greater storage of semantic memory.^22^

Smith et al.^23^ concluded that the CONCEPT and MEM subscales, as well as the total score on DRS, display good psychometric properties. On the other hand, these authors stated that the ATT and CONST subscales should be carefully used, whereas reliability was not found for I/P. The lower influence of education on the MEM subtest in the present study reinforces its usefulness for diagnostic purposes. Lukatela et al.^5^ found that the MEM subscale has a predominant role in discriminating between different types of dementia.

Influence of age on the scale performance was not verified. Considering that many studies show the influence of this factor,^3^ ^,^ ^10^ ^,^ ^21^ the study results may be explained by the age group of the sample, from 45 to 64 years. The influence of sex was not found either, similar to other studies.^3^ ^,^ ^10^ ^,^ ^24^ However, there were many women in the sample, which may have limited this analysis.

Regarding IQ, this variable was included following the recommendation of Mitrushina et al. found in a guide for normative studies on neuropsychological tests.^25^ It consists in a way to verify the influence of a present ability on DRS results, in contrast to formal education, which the participants had often completed years ago. In the overall comments about the DRS, Strauss, Spreen, and Sherman stated that the scale has a relatively good concurrent validity, with good correlation with the Wechsler scales (memory and intelligence).^3^ The present study showed a close relationship between the total score and the WASI, reinforcing this evidence. On the other hand, this study found a similar influence of education level and IQ on the results. Considering the time and cost of including an intelligence scale, researchers should think about its usefulness. Some authors suggest the investigation of other abilities instead of IQ such as reading habit.^26^

The present study has the limitation of selecting healthy people from a hospital unit among healthcare professionals and patients’ caregivers. Multicenter studies should be further performed with a more representative sample of the Brazilian population.

Finally, the authors hope that normative data in the DRS presented in this study can support Brazilian clinicians and researchers in the great challenge posed by dementia diagnosis and follow-up.
